# Con?icted Identities and Art Therapy: Practices and Case Studies in Kolozsvar/Cluj-Napoca, Romania

**DOI:** 10.1192/j.eurpsy.2022.1407

**Published:** 2022-09-01

**Authors:** E. Chirilă

**Affiliations:** CONSILIUL JUDEŢEAN CLUJ Direcţia Generală de Asistenţă Socială şi Protecţia Copilului, Centrul Comunitar JudeŢean Complex De Servicii Sociale Comunitare Pentru Copii și Adulţi Cluj, Cluj-napoca , Romania

**Keywords:** art therapy, play /occupational therapy, multimedia technology, physical and metaphysical environmen, art therapy, play /occupational therapy/ multimedia technology/physical and metaphysical environmen

## Abstract

**Introduction:**

. Cluj-Napoca in Transylvania, Romania, has a historically multiethnic population who maintain their language-based cultural identities. In order to harmonize interethnic relations in our multicultural society, art-therapeutical methods depend on the need to establish a sensitive relationship between the cultural horizon of individuals, thus increasing self-confidence, tolerance, resilience.

**Figure:**
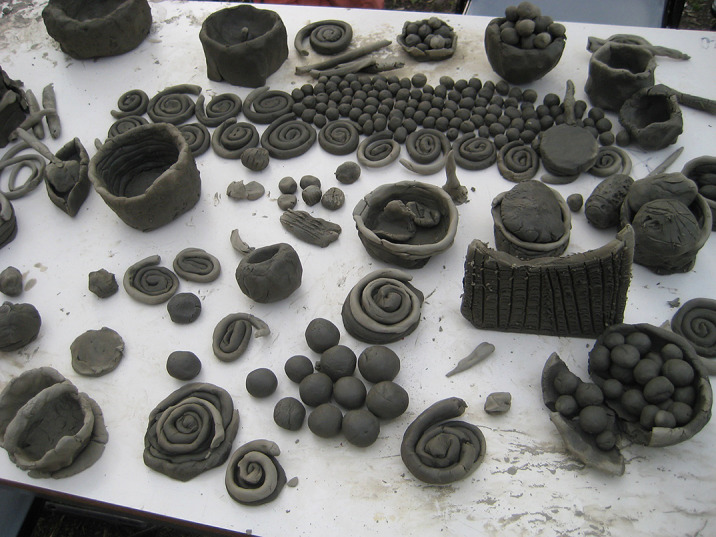

**Objectives:**

The objectives are : to develop social skills, which facilitate the social and professional integration of children and adolescents belonging to ethnic groups living together, including those with disabilities.

**Figure:**
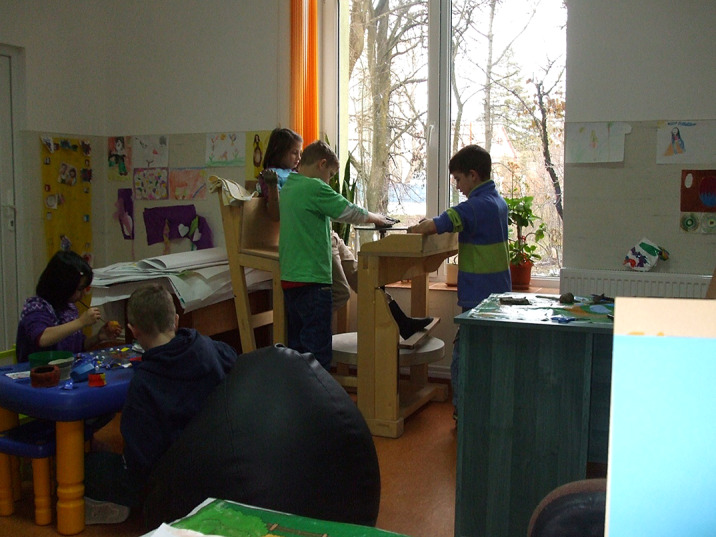

**Figure:**
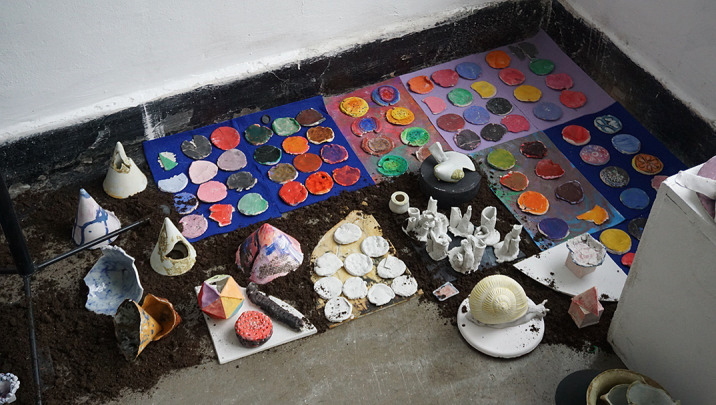

**Methods:**

Clinical art therapy have unfolded within interdisciplinary teams: a neuropsychiatry doctor, a psychologist, a pedagogue, a social worker, an art therapist– each one having a specialized role. A medical project was transformed into an artistic project: E xperimenting with complex relationships: shape of the human body – shape of man-made objects and the creation of personal shapes conduct to harmonize interethnic relations in a multicultural place.

**Results:**

Focus on several objectives: - practicing the abilities to express one’s feelings - the consolidation of self-respect and of confidence - the training of empathy - the development of personal problem and conflict solving strategies -the breaking through the emotional blockages - the improvement of cognitive abilities -the release of tension, frustrations, anxieties, stress -the development of social skills

**Conclusions:**

Benefits arise from experiences based in artistic creativity: materializing ideas and coping with unexpected outcomes.

**Disclosure:**

No significant relationships.

